# Impact of COVID-19 pandemic on birth outcomes: A retrospective cohort study in Nanjing, China

**DOI:** 10.3389/fpubh.2022.923324

**Published:** 2022-07-18

**Authors:** Juan Wen

**Affiliations:** Nanjing Maternity and Child Health Care Institute, Women's Hospital of Nanjing Medical University (Nanjing Maternity and Child Health Care Hospital), Nanjing, China

**Keywords:** preterm birth, birth weight, COVID-19 pandemic, retrospective cohort, China

## Abstract

**Introduction:**

The coronavirus disease 2019 (COVID-19) pandemic have significantly affected health care systems and daily wellbeing. However, the indirect impacts of the pandemic on birth outcomes are not fully understood. We aimed to examine whether the pandemic altered risk of adverse birth outcomes.

**Methods:**

This retrospective cohort study included all singleton births during 2016–2020 identified in Women's Hospital of Nanjing Medical University. We compared birth outcomes during COVID-19 pandemic (January–December 2020) with before the pandemic (January–December 2016–2019) using Logstic regression adjusted for confounders.

**Results:**

A total of 19,792 and 92,750 births occurred during and before the pandemic, respectively. Maternal characteristics were similar between groups, except maternal age was higher in pandemic cohort. We observed a reduction in preterm birth (PTB, <37 weeks) during the pandemic [5.9 vs. 5.1%, OR (95%CI) = 0.86 (0.80, 0.92)], but the difference disappeared after multivariable adjustment [adjusted OR (95%CI) = 1.02 (0.94, 1.11)]. Moreover, full term infants born during the pandemic had lower birth weights than those born before the pandemic [adjusted β (95% CI) = −17.4 (−23.9, −10.8)]. Consistently, the risks of low birthweight (LBW, <2,500 g) and small for gestational age (SGA, < P_10_) were increased [LBW: adjusted OR (95%CI) = 1.13 (1.02, 1.24); SGA: adjusted OR (95%CI) = 1.11 (1.02, 1.21)], and the risks of macrosomia (≥4,000 g) and large for gestational age (LGA, ≥P_90_) were decreased in the pandemic cohort [macrosomia: adjusted OR (95%CI) = 0.82 (0.77, 0.88); LGA: adjusted OR (95%CI) = 0.73 (0.69, 0.77)].

**Conclusion:**

In this study, we observed no change in preterm birth and a decrease in birth weight of full term infants during the pandemic in Nanjing, China.

## Introduction

The coronavirus disease 2019 (COVID-19) pandemic has resulted in substantial morbidity and mortality, and also created a profound impact on health care systems, social functioning and daily wellbeing (1, 2). To restrict the spread of the disease, countries imposed national or regional lockdown, which consisted of multiple restrictions measures including stay-at-home orders, working at home, health care disruption, schools and shops closure except for emergency services (3, 4). The widespread lockdown is unprecedented, and the impact on human physical and mental health is not fully understood (5).

Previous studies have found that the COVID-19 pandemic may have influenced obstetric interventions and birth outcomes due to disruption of maternal and neonatal health services and massive stress from psychsocial and economic consequences of the pandemic (6, 7). There is overwhelming evidence that the COVID-19 pandemic has led to the emergence and exacerbation of mental health issues such as stress, anxiety and depression, although maternal workload and work stress have declined during the pandemic (8, 9). Most attention has been paid to the impact of the pandemic on preterm birth (PTB), but with inconsistent results. Reductions in PTB rates during COVID-19 pandemic compared with before pandemic have been reported in many countries, such as Australia (10–12), the United States (13–15), Israel (16), the Netherlands (17), Denmark and Ireland (18–20), while studies in China, Sweden and Spain have not found such changes (21–23). In a recent meta-analysis, PTB was not significantly changed overall but was decreased in high-income countries (24). In another meta-analysis, this reduction was noted only in unadjusted estimates and in single-center studies, raising the possibility of publication bias and need for further research (25). Birth weight, a sensitive indicator of the intrauterine growth, is another concern regarding the impacts of the pandemic (4). Recent studies showed only a marginal increase of 17 g in mean birth weight during the pandemic compared to before pandemic (25). In contrast, other studies observed no significant difference in low birth weight (<2,500 g) associated with the pandemic (24). The COVID-19 lockdown and population response measures, as well as risk factors for adverse birth outcomes, vary from region to region, which may partly explain the differences between studies (26).

In China, the first case of COVID-19 was detected on Dec 8, 2019, and the first death caused by COVID-19 was confirmed on January 9, 2020. As a result of additional cases being detected, the Chinese government announced a national lockdown on January 25, 2020, to practice social distancing. As the epidemic was gradually brought under control, China entered the phase of regular epidemic prevention and control on April 29, 2020. Until now, China is still in this phase in which the epidemic is sporadic, and there are occasionally small clusters of local epidemics with regional lockdown ongoing. Suspicion is widespread that China's response to COVID-19 may have had an indirect impact on pregnant women and their babies. In a retrospective analyses conducted in Beijing, China, the risk of premature rupture of membranes and fetal distress was reported to increase by 11 and 14%, respectively, during the COVID-2019 pandemic (21). This retrospective cohort study was designed to compare birth outcomes in two populations of pregnant women in Nanjing, China: one delivered during COVID-19 pandemic (January–December 2020) and the other before the pandemic (January–December 2016–2019).

## Methods

### Study design and population

This is a retrospective cohort study including all pregnancy women who delivered at Women's Hospital of Nanjing Medical University in 2016–2020. The Women's Hospital of Nanjing Medical University is the largest maternity hospital in Jiangsu province, China. After excluding women delivered before 28 weeks of gestation, multiple gestation pregnancies and those with missing outcome data, a total of 112,542 live births were included in the data analysis. Two cohorts were created: women who delivered between January, 2020 (when COVID-19 cases first became widely reported in Wuhan, China), and December, 2020, were considered as the pandemic cohort, and women who delivered between January, 2016 and December, 2019 (4 years before the pandemic), were considered as the pre-pandemic cohort. Women in pandemic cohort were all negative for COVID-19.

The institutional review board of Women's Hospital of Nanjing Medical University approved the study (2020KY-011) and waived the requirement for informed consent because of the retrospective design.

### Data collection and outcomes definition

All maternal and neonatal information was obtained from Hospital Information System (HIS) Database. Data were collected from standardized clinical forms and hospital records after maternity discharge to form the research database. All data were extracted and cleaned by using Natural Language Processing technique (27). Maternal characteristics of all pregnant women were firstly extracted, including maternal age (year), height (cm), intrapartum weight (kg), parity, gestational week at birth, singleton or multiple gestation, menstrual cycle (21–35 days, 36 days- or irregularity), abnormal pregnancy history and pregnancy conceived with assisted reproductive technology (ART). Maternal age was divided into five groups: <25, 25–29, 30–34, 35–39, ≥40 years. Intrapartum body mass index (BMI, kg/m^2^) was calculated as maternal intrapartum weight divided by the square of height, and classified into four groups: <25, 25–29.9, 30–34.9, ≥35 kg/m^2^. Parity did not include this pregnancy and was divided into 0 (nulliparae) and ≥1 (multiparae). Abnormal pregnancy history refers to a history of spontaneous abortion, fetal malformation, or stillbirth. An ART pregnancy was defined as one conceived following intracytoplasmic sperm injection, *in vitro* fertilization and embryo transplantation, ovulation induction, gamete intra-fallopian transfer, or artificial insemination.

The birth outcomes of interest in this study were PTB, low birthweight or macrosomia, and small or large for gestational age. We calculated PTB (<37 weeks of gestation) and very PTB (VPTB, <32 weeks of gestation) based on the clinician's best estimate of gestational age. We then used the HIS Database to obtain data on birthweight (g). Low birthweight (LBW) was defined as 1,500–2,500 g, very LBW (VLBW) as 1,000–1,500 g, and macrosomia as more than 4,000 g. Small for gestational age (SGA) was defined as birthweight less than the 10th percentile, very SGA (VSGA) as birthweight less than the 3th percentile, and large for gestational age (LGA) as birthweight greater than the 90th percentile by gestational week at birth (28–30).

### Statistical analyses

In this article, we compared maternal characteristics and birth outcomes between the pandemic and prepandemic cohort. Continuous variables were described as mean and standard deviation (*x* ± *s*), and compared between the two cohorts by Student's *t*-test, while categorical variables were displayed as frequency (percentage) and compared by χ^2^-test. The impacts of pandemic on each birth outcome were evaluated by logistic regression analysis, and the effect of pandemic on birthweight was assessed by linear regression analysis in full term infants only. In the adjusted models, maternal age, intrapartum BMI and parity were controlled. The crude and adjusted odds ratio (OR) or β with 95% confidence intervals (95%CI) for birth outcomes and birthweight were calculated. To further analyse the possible impacts of maternal age, BMI and parity on the association between pandemic and birth outcomes, comparisons (pandemic vs. pre-pandemic cohort) were also stratified by maternal age groups (<30 and ≥30 years), intrapartum BMI groups (<30 and ≥30 kg/m^2^) and parity (nulliparae and multiparae). Statistical analyses were performed using SPSS v25.0, and the reported statistical significance levels were all two-sided, with *P* < 0.05 considered significant.

## Results

A total of 112,542 women were included in the retrospective analysis, of whom 92,750 women delivered during 2016–2019 as pre-pandemic cohort, and 19,792 women delivered in 2020 as pandemic cohort. Maternal characteristics across years and across cohorts are summarized in Table 1. The maternal characteristics (intrapartum BMI, parity, menstrual cycle, abnormal pregnancy history and ART pregnancy) were similar between the pre-pandemic and pandemic cohort, except maternal age was significantly higher in pandemic cohort as compared with pre-pandemic cohort (30.3 ± 3.9 years vs. 29.9 ± 4.0 years, *P* < 0.001).

**Table 1:** Maternal characteristics between the pre-pandemic and pandemic cohort.

**Maternal characteristics**	**2016**	**2017**	**2018**	**2019**	**Pre-pandemic cohort (2016–2019)**	**Pandemic cohort (2020)**	P*
Total births	23,108	23,017	23,280	23,345	92,750	19,792	-
Maternal age (years)	29.7 ± 3.8	30.0 ± 4.1	30.0 ± 4.0	30.0 ± 3.9	29.9 ± 4.0	30.3 ± 3.9	<0.001
Intrapartum BMI (kg/m^2^)	26.7 ± 3.1	26.8 ± 3.1	26.7 ± 3.1	26.7 ± 3.2	26.7 ± 3.1	26.7 ± 3.2	0.982
Multiparae	6,252 (27.1)	7,789 (33.8)	7,546 (32.4)	7,117 (30.5)	28,704 (30.9)	6,077 (30.7)	0.501
Irregular menstrual cycle	1,674 (7.3)	1,564 (6.8)	1,747 (7.5)	1,560 (6.7)	6,545 (7.1)	1,473 (7.4)	0.068
Abnormal pregnancy history	3,257 (14.1)	3,531 (15.4)	3,609 (15.5)	3,260 (14.0)	13,657 (14.7)	2,965 (15.0)	0.386
ART pregnancy	910 (3.9)	1,173 (5.1)	1,339 (5.8)	1,457 (6.2)	4,879 (5.3)	1,108 (5.6)	0.055
Gestational week	38.8 ± 1.7	38.8 ± 1.7	38.8 ± 1.7	38.9 ± 1.6	38.8 ± 1.7	38.9 ± 1.6	<0.001

*Continuous variables are shown as mean and standard deviation ($\bar{x}$ ± s), and compared between the two study cohorts by Student's t-test, while categorical variables are displayed as frequency (percentage) and compared by χ^2−^test. P values in bold are significant*.

^*^Comparison between the pre-pandemic and pandemic cohort.

*BMI, body mass index; ART, assisted reproductive technology*.

In terms of birth outcomes, PTB was the primary outcome. We first observed a significant increase in gestational week at birth in the pandemic cohort compared to the pre-pandemic cohort (38.9 ± 1.6 vs. 38.8 ± 1.7 weeks, *P* < 0.001) (Table 1), accompanied by a significant decrease in the rate of PTB (<37 weeks, 5.1 vs. 5.9%, *P* < 0.001), but no significant change in the rate of VPTB (<32 weeks). Moreover, the birth weight of full term infants in pandemic cohort was lighter than that in pre-pandemic cohort (3,392.5 ± 390.5 vs. 3,407.5 ± 396.0 g, *P* < 0.001). After classifying the birth weight, we observed macrosomia (≥4,000 g, 6.2 vs. 7.2%, *P* < 0.001) and LGA (≥P_90_, 9.6 vs. 12.2%, *P* < 0.001) rates were lower, and SGA (< P_10_, 4.0 vs. 3.7%, *P* = 0.047) rate was slightly higher in the pandemic cohort than in pre-pandemic cohort (Table 2).

**Table 2:** The association between COVID-19 pandemic and birth outcomes.

**Birth outcomes**	**Pre-pandemic cohort**	**Pandemic cohort**	**Pre-pandemic cohort**	**Pandemic cohort**			
				**OR/**β **(95%CI)**	***P***	**Adjusted OR/**β (95%CI)*	P*
PTB (<37 weeks)	5,472 (5.9)	1,011 (5.1)	1.0 (ref)	0.86 (0.80, 0.92)	**<0.001**	1.02 (0.94, 1.11)	0.666
VPTB (<32 weeks)	755 (0.8)	161 (0.8)	1.0 (ref)	1.00 (0.84, 1.19)	0.994	1.06 (0.87, 1.28)	0.581
Birth weight (g)	3,407.5 ± 396.0	3,392.5 ± 390.5	1.0 (ref)	−15.03 (−21.26, −8.81)	<0.001	−17.36 (−23.89, −10.83)	<0.001
LBW (<2,500 g)	3,581 (3.9)	753 (3.8)	1.0 (ref)	0.99 (0.91, 1.07)	0.708	1.13 (1.02, 1.24)	**0.015**
VLBW (<1,500 g)	521 (0.6)	105 (0.5)	1.0 (ref)	0.94 (0.77, 1.17)	0.592	0.99 (0.78, 1.25)	0.913
Macrosomia (≥4,000 g)	6,638 (7.2)	1,229 (6.2)	1.0 (ref)	0.86 (0.81, 0.92)	**<0.001**	0.82 (0.77, 0.88)	**<0.001**
SGA (< P_10_)	3,451 (3.7)	795 (4.0)	1.0 (ref)	1.08 (1.00, 1.17)	**0.047**	1.11 (1.02, 1.21)	**0.019**
VSGA (< P_3_)	927 (1.0)	206 (1.0)	1.0 (ref)	1.04 (0.90, 1.21)	0.596	1.09 (0.92, 1.28)	0.329
LGA (≥P_90_)	11,278 (12.2)	1,891 (9.6)	1.0 (ref)	0.76 (0.73, 0.80)	**<0.001**	0.73 (0.69, 0.77)	**<0.001**

^*^Logistic/linear regression analyses adjusted for maternal age, intrapartum BMI and parity; logistic regression analyses for birth outcomes except for birth weight [values are OR (95% CI)]; linear regression analyses for birth weight in full term infants only [values are β (95% CI)]. P values in bold are significant.

*BMI, body mass index; PTB, preterm birth; VPTB, very preterm birth; LBW, low birthweight; VLBW, very low birthweight; SGA, small for gestational age; VSGA, very small for gestational age; LGA, large for gestational age*.

Univariate logistic regression analysis showed that the risk of PTB (<37 weeks) was significantly decreased in the pandemic cohort as compared with pre-pandemic cohort [OR (95%CI) = 0.86 (0.80, 0.92), *P* < 0.001], but the significant difference disappeared after adjustment for multiple factors [adjusted OR (95%CI) = 1.02 (0.94, 1.11), *P* = 0.666], suggesting that confounding factors such as maternal age, BMI and parity play important roles in the occurrence of PTB. In addition, multivariable linear regression analysis showed that full term infants born during the pandemic had lower birth weights than those born before the pandemic [adjusted β (95% CI) = −17.36 (−23.89, −10.83), *P* < 0.001]. Consistently, in multivariable logistic regression model, the risk of LBW (<2,500 g) and SGA (< P_10_) were significantly increased [LBW: adjusted OR (95%CI) = 1.13 (1.02, 1.24), *P* = 0.015; SGA: adjusted OR (95%CI) = 1.11 (1.02, 1.21), *P* = 0.019], and the risk of macrosomia (≥4,000 g) and LGA (≥P_90_) were significantly decreased in the pandemic cohort [macrosomia: adjusted OR (95%CI) = 0.82 (0.77, 0.88), *P* < 0.001; LGA: adjusted OR (95%CI) = 0.73 (0.69, 0.77), *P* < 0.001] (Table 2).

The impact of COVID-19 pandemic on birth outcomes was also evaluated by stratifying on maternal age, intrapartum BMI and parity (Tables 3–5). Similar association strengths were shown between most subgroups (heterogeneity test: *P* > 0.10). Interestingly, a more prominent effect of the pandemic on LGA (≥P_90_) risk was observed among women with BMI <30 kg/m^2^ [adjusted OR (95%CI) = 0.71 (0.66, 0.76)] compared with that in women with BMI >30 kg/m^2^ [adjusted OR (95%CI) = 0.81 (0.72, 0.90); Heterogeneity test: *P* = 0.050]. We also observed a stronger effect of the pandemic on macrosomia occurrence in nulliparaes [adjusted OR (95%CI) = 0.77 (0.71, 0.84)] as compared with that in multiparaes [adjusted OR (95%CI) = 0.90 (0.81, 1.01); Heterogeneity test: *P* = 0.028].

**Table 3:** Stratified analyses on the association between COVID-19 pandemic and birth outcomes by maternal age.

**Birth outcomes**	**Pre-pandemic cohort**	**Pandemic cohort**			
		**Maternal age**<**30 years**		**Maternal age** ≥**30 years**	
		**Adjusted OR/**β **(95%CI)**	***P***	**Adjusted OR/**β **(95%CI)**	***P***
PTB (<37 weeks)	1.0 (ref)	1.05 (0.92, 1.20)	0.480	0.99 (0.88, 1.11)	0.804
VPTB (<32 weeks)	1.0 (ref)	1.04 (0.78, 1.40)	0.789	1.06 (0.82, 1.36)	0.682
Birth weight (g)	1.0 (ref)	−22.63 (−31.99, −13.27)	<0.001	−11.73 (−20.89, −2.57)	0.012
LBW (<2,500 g)	1.0 (ref)	1.04 (0.89, 1.20)	0.645	1.19 (1.05, 1.35)	0.007
VLBW (<1,500 g)	1.0 (ref)	1.00 (0.70, 1.44)	0.987	0.96 (0.71, 1.32)	0.814
Macrosomia (≥4,000 g)	1.0 (ref)	0.78 (0.70, 0.86)	<0.001	0.85 (0.78, 0.93)	0.001
SGA (< P_10_)	1.0 (ref)	1.09 (0.97, 1.23)	0.163	1.12 (0.99, 1.26)	0.076
VSGA (< P_3_)	1.0 (ref)	1.02 (0.80, 1.30)	0.886	1.13 (0.90, 1.42)	0.281
LGA (≥P_90_)	1.0 (ref)	0.71 (0.65, 0.78)	<0.001	0.74 (0.69, 0.80)	<0.001

*Logistic/linear regression analyses adjusted for intrapartum BMI and parity; logistic regression analyses for birth outcomes except for birth weight [values are OR (95% CI)]; linear regression analyses for birth weight in full term infants only [values are β (95% CI)]*.

*BMI, body mass index; PTB, preterm birth; VPTB, very preterm birth; LBW, low birthweight; VLBW, very low birthweight; SGA, small for gestational age; VSGA, very small for gestational age; LGA, large for gestational age*.

**Table 4:** Stratified analyses on the association between COVID-19 pandemic and birth outcomes by intrapartum BMI.

**Birth outcomes**	**Pre-pandemic cohort**	**Pandemic cohort**			
		**Intrapartum BMI**<**30 kg/m**^2^		**Intrapartum BMI** ≥**30 kg/m**^2^	
		**Adjusted OR/**β **(95%CI)**	***P***	**Adjusted OR/**β **(95%CI)**	***P***
PTB (<37 weeks)	1.0 (ref)	1.00 (0.91, 1.10)	0.969	1.14 (0.92, 1.40)	0.236
VPTB (<32 weeks)	1.0 (ref)	1.06 (0.86, 1.31)	0.569	1.03 (0.62, 1.71)	0.905
Birth weight (g)	1.0 (ref)	−17.32 (−24.40, −10.25)	<0.001	−27.98 (−47.45, −8.51)	0.005
LBW (<2,500 g)	1.0 (ref)	1.10 (0.99, 1.22)	0.076	1.29 (1.02, 1.62)	0.035
VLBW (<1,500 g)	1.0 (ref)	1.02 (0.79, 1.32)	0.856	0.82 (0.44, 1.52)	0.529
Macrosomia (≥4,000 g)	1.0 (ref)	0.80 (0.74, 0.87)	<0.001	0.88 (0.78, 0.99)	0.037
SGA (< P_10_)	1.0 (ref)	1.09 (1.00, 1.20)	0.059	1.36 (1.05, 1.76)	0.021
VSGA (< P_3_)	1.0 (ref)	1.14 (0.96, 1.37)	0.136	0.79 (0.50, 1.27)	0.332
LGA (≥P_90_)	1.0 (ref)	0.71 (0.66, 0.76)	<0.001	0.81 (0.72, 0.90)	<0.001

*Logistic/linear regression analyses adjusted for maternal age and parity; logistic regression analyses for birth outcomes except for birth weight [values are OR (95% CI)]; linear regression analyses for birth weight in full term infants only [values are β (95% CI)]*.

*BMI, body mass index; PTB, preterm birth; VPTB, very preterm birth; LBW, low birthweight; VLBW, very low birthweight; SGA, small for gestational age; VSGA, very small for gestational age; LGA, large for gestational age*.

**Table 5:** Stratified analyses on the association between COVID-19 pandemic and birth outcomes by parity.

**Birth outcomes**	**Pre-pandemic cohort**	**Pandemic cohort**			
		**Nulliparae**		**Multiparae**	
		**Adjusted OR/**β **(95%CI)**	***P***	**Adjusted OR/**β **(95%CI)**	***P***
PTB (<37 weeks)	1.0 (ref)	1.07 (0.96, 1.19)	0.233	0.95 (0.82, 1.09)	0.446
VPTB (<32 weeks)	1.0 (ref)	1.10 (0.85, 1.43)	0.453	1.01 (0.76, 1.35)	0.937
Birth weight (g)	1.0 (ref)	−20.21 (−28.00, −12.41)	<0.001	−9.89 (−21.87, 2.10)	0.106
LBW (<2,500 g)	1.0 (ref)	1.17 (1.04, 1.31)	0.012	1.07 (0.91, 1.26)	0.400
VLBW (<1,500 g)	1.0 (ref)	1.09 (0.81, 1.47)	0.554	0.86 (0.58, 1.27)	0.434
Macrosomia (≥4,000 g)	1.0 (ref)	0.77 (0.71, 0.84)	<0.001	0.90 (0.81, 1.01)	0.072
SGA (< P_10_)	1.0 (ref)	1.10 (1.00, 1.21)	0.057	1.15 (0.95, 1.39)	0.142
VSGA (< P_3_)	1.0 (ref)	1.08 (0.89, 1.31)	0.451	1.13 (0.81, 1.56)	0.472
LGA (≥P_90_)	1.0 (ref)	0.70 (0.64, 0.75)	<0.001	0.77 (0.71, 0.84)	<0.001

*Logistic/linear regression analyses adjusted for maternal age and intrapartum BMI; logistic regression analyses for birth outcomes except for birth weight [values are OR (95% CI)]; linear regression analyses for birth weight in full term infants only [values are β (95% CI)]*.

*BMI, body mass index; PTB, preterm birth; VPTB, very preterm birth; LBW, low birthweight; VLBW, very low birthweight; SGA, small for gestational age; VSGA, very small for gestational age; LGA, large for gestational age*.

## Discussion

In response to the COVID-19 crisis, China enforced a lockdown in 2020 that restricted movement within the country. We used electronic medical records from HIS Database to evaluate birth outcomes during and before COVID-19 pandemic. A reduction in PTB rate was observed during the pandemic, but the change disappeared after multivariable adjustment. Moreover, full term infants born during the pandemic had lower birth weights than those born before the pandemic.

In the present study, the risk of PTB <37 weeks did not change significantly during the COVID-19 pandemic, which was supported by two recent meta-analysis (24, 25). However, studies conducted in high-income countries or in single-center suggested the pandemic would reduce the risk of PTB (24, 25). The researchers have proposed that COVID-19 related lockdown may cause socio-environmental and behavioural modifications, including maternal workload reduction, improved air quality, reduced maternal non-COVID-19 related infections, reductions in physical activity and better nutritional support, thus paly a role in pregnancy prolongation and exert a benefical impact on preterm birth (3, 5, 26, 31). On the other hand, several recent studies have shown that COVID-19 pandemic related stressors and quarantine measures have exacerbated perinatal anxiety and depression (8, 9). Stress, worries and anxieties during pregnancy are often associated with preterm birth (32). Moreover, COVID-19 lockdown may result in a reduction in antenatal care and fetal surveillance. Therefore, the impact of the pandemic on preterm birth is a double-edged sword. For the risk of VPTB (<32 weeks), we also found no significant changes during the pandemic, which was in accordance with previous meta-analysis and subgroup analyses (24, 25).

The decrease in mean birth weight during the pandemic in this study was inconsistent with the findings of several previous studies (16, 33, 34). Our finding of decreased birth weight should be interpreted in the context of the COVID-19 mitigation strategy used in China. During the pandemic, in response to social distancing policies, pregnant women may eat less frequently in restaurants, usually rich in fats, sugars, and salt, which may be beneficial to the control of weight gain during pregnancy to some extent. Moreover, anxiety and stress caused by the pandemic are also contributing factors. Studies have suggested that stressful life events are associated with decreases in birth weight and thus, increased risks of LBW (32, 35). COVID-19 is not only a pandemic and a global health crisis, but also a psychosocial and economic disaster. Economic crises, such as the 2008 financial crisis, have also led to declines in mean birth weight in countries that have been particularly affected (36–38). Since the economic impact of the COVID-19 pandemic is comparable to that of the 2008 financial crisis, the COVID-19 pandemic could have a similar impact on intrauterine development and birth outcomes. In this study, the rates of macrosomia (≥4,000 g) and LGA (≥P_90_) decreased, and the rate of SGA (< P_10_) slightly increased during the COVID-19 pandemic. These findings are in accordance with the observations during the 2008 financial crisis. Interestingly, several studies have shown that the COVID-19 pandemic has no significant impact on birth weight (4, 7, 39–42).

We then compared our results with a similar study also conducted in China (21). The authors of this study conducted retrospective analyses on two cohorts comprising 7,699 pregnant women in Beijing, China, and compared pregnancy outcomes between the pre-COVID-2019 cohort and the COVID-2019 cohort. They uncovered no associations between the COVID-19 pandemic and preterm birth, low birth weight and macrosomia (*P* > 0.05). But they found the risk of premature rupture of membranes and fetal distress was increased by 11 and 14%, respectively, during the COVID-2019 pandemic. Explanations for these results may be related to the pandemic mitigation measures and population responses in each region.

The main advantages of this study were the large sample size of pregnancies, which enabled us to perform further subgroup analysis with enough power, and the quality of the data obtained from HIS Database with Natural Language Processing technique is high. Moreover, we conducted a manual comparison of some data as quality control to ensure the reliability of extracted data. Nevertheless, this study had some limitations. First, the population we studied was limited to one city in eastern China (Nanjing). So caution should be taken when generalizing our findings to other regions. Second, we did not collect more detailed information, such as pre-pregnancy BMI and weight gain during pregnancy, which were not adjusted in our analysis, and might result in over-estimation of the effect sizes in this study. Third, the retrospective design in this study could not assess the direct impact of COVID-19 pandemic on birth outcomes. All these potential limitations should be considered when interpreting the results.

Although this retrospective study suggested that COVID-19 pandemic was associated with birth weight, the link between the pandemic and birth outcomes remains ambiguous. Further research will clarify whether changes in birth outcomes are related to changes in health-related behaviors during the pandemic. There is also a need to assess the availability of maternal and newborn health services. Research in these areas will allow us to draw up plans and allocate resources effectively for immediate care after the pandemic and for future health system crises.

## Data availability statement

The original contributions presented in the study are included in the article/supplementary material, further inquiries can be directed to the corresponding author.

## Ethics statement

The studies involving human participants were reviewed and approved by the Institutional Review Board of Women's Hospital of Nanjing Medical University (2020KY-011). Written informed consent for participation was not required for this study in accordance with the national legislation and the institutional requirements.

## Author contributions

JW did data collection, performed the data analysis, and written the manuscript.

## Funding

This work was supported by Jiangsu Provincial Key Research and Development Program (BE2020626).

## Conflict of interest

The author declares that the research was conducted in the absence of any commercial or financial relationships that could be construed as a potential conflict of interest.

## Publisher's note

All claims expressed in this article are solely those of the authors and do not necessarily represent those of their affiliated organizations, or those of the publisher, the editors and the reviewers. Any product that may be evaluated in this article, or claim that may be made by its manufacturer, is not guaranteed or endorsed by the publisher.
